# sB7H3 in Children with Acute Appendicitis: Its Diagnostic Value and Association with Histological Findings

**DOI:** 10.1155/2020/2670527

**Published:** 2020-09-01

**Authors:** Xiaochen Du, Yan Chen, Jie Zhu, Zhenjiang Bai, Jun Hua, Ying Li, Haitao Lv, Guangbo Zhang

**Affiliations:** ^1^Department of Emergency and Intensive Care Unit, Children's Hospital of Soochow University, Suzhou, Jiangsu Province 215025, China; ^2^Department of Emergency, Children's Hospital of Wujiang District, Suzhou, Jiangsu Province 215200, China; ^3^Department of General Surgery, The First Affiliated Hospital of Soochow University, Suzhou, Jiangsu Province 215006, China; ^4^Department of Pediatric Surgery, Children's Hospital of Soochow University, Suzhou, Jiangsu Province 215025, China; ^5^Department of Cardiology, Children's Hospital of Soochow University, Suzhou, Jiangsu Province 215025, China; ^6^Institute of Clinical Immunology, The First Affiliated Hospital of Soochow University, Suzhou, Jiangsu Province 215006, China

## Abstract

**Background:**

Several efforts have been made to find out a valuable marker to assist the diagnosis and differentiation of gangrenous/perforated appendicitis. We aimed to determine the diagnostic capacity of soluble B7H3 (sB7H3) in acute appendicitis (AA) and its accuracy as a predictor of the severity of appendicitis.

**Methods:**

182 children were allocated into four groups as follows: control group (CG, 90), simple appendicitis (SA, 12), purulent appendicitis (PA, 49), and gangrenous appendicitis (GA, 31). Prior to appendectomy, blood was collected and sent for analysis of routine examination and cytokines (sB7H3 and TNF-*α*). We compared values of all measured parameters according to histological findings. Furthermore, we assigned AA patients into the nonperforated appendicitis group and the perforated appendicitis group. The diagnostic effects of significant markers were assessed by ROC curves.

**Results:**

Only the levels of CRP, FIB, and sB7H3 had a remarkable rising trend in AA-based groups, while differences in the levels of CRP and FIB between simple appendicitis and purulent appendicitis were not statistically significant. In addition, sB7H3 was found as the only marker in children with AA, which was markedly associated with the degree of histological findings of the appendix. Furthermore, sB7H3 had a high diagnostic value in predicting AA and complex appendicitis (PA+GA) in children. However, the diagnostic performance of sB7H3 for distinguishing PA from GA was not remarkable. Additionally, only the levels of CRP and sB7H3 were statistically different between the nonperforated appendicitis group and the perforated appendicitis group. The diagnostic performance of CRP and sB7H3 could not merely predict perforation of AA in children; however, the diagnostic performance was improved after combination.

**Conclusions:**

sB7H3 could be used as a valuable marker to predict the presence of AA and complex AA in children. However, the diagnostic value of sB7H3 to predict gangrenous/perforated appendicitis was not found to be remarkable. The combination of sB7H3 and CRP might improve the prediction of perforated appendicitis.

## 1. Introduction

Acute abdominal pain is one of the frequent chief complaints of children; acute appendicitis (AA) is the most common surgical emergency in the pediatric population [1]. It has been estimated that appendectomy was annually carried out on 72,000 children in the United States [2]. Compared with adults, performing a clinical diagnosis of appendicitis in children is often difficult due to their incomplete history and atypical symptoms.

Although controversy exists in the literature about the exact clinical classification, appendicitis can be classified as “simple” or “complicated.” Complicated appendicitis is associated with a variety of potentially serious complications like generalized peritonitis, abscess formation, and small bowel obstruction. Furthermore, a delayed diagnosis and surgery for AA are associated with increased perforation rate for both children and adults [3]. In addition, the perforation of the inflamed appendix may result in peritonitis or intra-abdominal abscess formation. Simultaneously, overdiagnosis may result in expensive interhospital transfers and unnecessary surgery.

White blood cell (WBC) count and C-reactive protein (CRP) are frequently used by surgeons in emergency departments to diagnose AA, especially in children, women at the age of fertility, and elderly patients when diagnosis is difficult. Studies on the diagnostic value of WBC and CRP for diagnosing appendicitis in children have reported contradictory results [4, 5]. It has been demonstrated that novel inflammatory biomarkers, such as calprotectin, lactoferrin, high-mobility group protein B1 (HMGB1), and hepcidin, appear to be promising for the diagnosis of suspected appendicitis [6, 7]. However, further exploration of these markers, as well as potential others, needs to be conducted [8].

Additionally, B7-H3, a new member of the B7 superfamily, acts as both a T cell costimulator and coinhibitor. The expression of B7-H3 protein can be induced by inflammatory cytokines, thereby playing a pivotal role in the regulation of T cell-mediated immune response [9]. Our previous studies have identified that circulating B7H3 levels in the cerebrospinal fluid (CSF) and plasma of children with bacterial meningitis are helpful markers to differentiate bacterial from aseptic meningitis, and circulating B7H3 level was demonstrated to be useful in evaluating the intensity of the infectious inflammatory process in the central nervous system of children [10]. Furthermore, we reported that patients diagnosed with sepsis, in contrast to healthy individuals, exhibited significant levels of raised plasma sB7H3 and that level correlated with the clinical outcome [11]. However, to date, no previous studies have assessed an association between sB7H3 level and AA.

Thus, the main aim of this prospective single-center study was to determine the diagnostic capacity of sB7H3 in pediatric patients with AA. Furthermore, the accuracy of sB7H3 as a predictor of the severity of appendicitis was assessed.

## 2. Materials and Methods

### 2.1. Study Population

A total of 92 children suspicious of having AA who were admitted to Children's Hospital of Soochow University (Suzhou, China) and underwent open or laparoscopic appendectomies between April 2015 and October 2015 were enrolled in the present study. Among them, 62 cases were male (Table 1). Included children aged at the range of 11 months and 14 years with continuous pain in the lower right abdomen and tenderness in the lower right abdomen who were highly suspicious of having AA. The diagnosis was conducted on the basis of pathological findings. Patients with symptoms who improved after conservative treatment, chronic appendicitis, and normal appendix were excluded.

**Figure 1:**
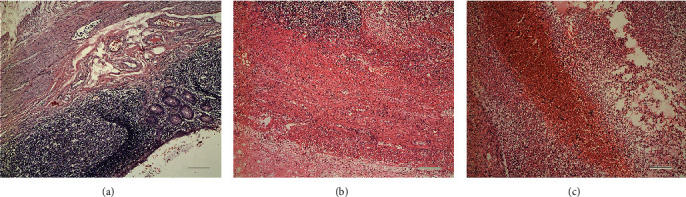
Typical histology of acute appendicitis at different stages (HE ×200). (a) Simple appendicitis: dilated and congested small blood vessels in the appendix wall, hyperplasia of mucosal lymphoid tissues, increased lymphoid follicles, enlarged germinal center, and obvious leukocyte adhesion in the local small mucosa of the mucosa. (b) Purulent appendicitis: dilated and congested small blood vessels in the appendix wall, neutrophil infiltration in each layer of tissue, fibrinous exudate, and necrosis in local mucosal layer tissue. (c) Gangrenous appendicitis: dilated and congested small blood vessels in the appendix wall, neutrophil infiltration in each layer of tissue, fibrinous exudate and necrosis, and hemorrhagic necrosis in whole layer of tissues. Internal scale bar = 50 *μ*m.

In the present study, patients with AA were assigned to three groups based on the histological diagnosis: (a) simple appendicitis (SA) (*n* = 12), (b) purulent appendicitis (PA) (*n* = 49), and (c) gangrenous appendicitis (GA) (*n* = 31). The typical histology of AA at different stages was shown in Figure 1. Meanwhile, 90 nonemergency inguinal hernia patients, who were age and gender matched, without pain in the abdomen and respiratory symptom were taken as the control group (CG) into account during the same period. According to the operative notes, all patients with AA were allocated to the nonperforated appendicitis group (*n* = 71) and the perforated appendicitis group (*n* = 21).

### 2.2. Ethics and Consent

The present study was approved by the Ethics Committee of Children's Hospital of Soochow University (Suzhou, China), and the written informed consent was obtained from parents or guardians of the recruited children prior to their enrolment. All experiments and procedures were conducted in accordance with the Declaration of Helsinki.

### 2.3. Routine Examination Determinations

Prior to appendectomy, a peripheral blood was sampled at admission and sent for blood routine testing, in addition to analysis of liver function and fibrinogen (FIB) level. Additionally, 2 mL serum was collected and centrifuged. Plasma samples were harvested and stored at -80°C for further experiment of sB7H3 and tumor necrosis factor-*α* (TNF-*α*) levels.

### 2.4. Measurement of Plasma sB7H3 and TNF-*α* Levels

The level of TNF-*α* was measured by using an enzyme-linked immunosorbent assay (ELISA) kit (R&D Systems, Minneapolis, MN, USA). The sB7H3 analyses were determined by using enzyme-linked immunosorbent assay (ELISA) kits (Suzhou Xuguang Kexing Biological Technology Co. Ltd., Suzhou, China) as previously described [12].

### 2.5. Statistical Analysis

In the present study, statistical analysis was conducted by using SPSS 22.0 software (IBM, Armonk, NY, USA). Measured data were expressed as mean ± standard deviation (SD). Enumeration data were expressed as rate (%). Moreover, Student's *t*-test and Mann–Whitney *U* test were used for comparing normally distributed and nonnormally distributed data between the groups, respectively. For comparing more than two groups, one-way analysis of variance (ANOVA) was employed, in addition to the Kruskal-Wallis test if data were nonnormally distributed. A chi-square test was used for comparing the rates between the acute nonperforated appendicitis group and the perforated appendicitis group, and then, the multivariate logistic regression analysis was utilized for the statistically significant markers. The diagnostic efficiency of these markers was evaluated by receiver operating characteristic (ROC) curves, and the cutoff values and area under the ROC curve (AUROC) of these markers were determined. The statistical significance was set at a two-sided *P* value of 0.05.

## 3. Results

### 3.1. Demographic Data and Clinical Characteristics of Patients with AA

The demographic and clinical characteristics of patients with AA and CG are presented (Table 1). There was no difference in gender between the CG and the AA group (*P* > 0.05); the age of the AA group was slightly older than that of the CG (*P* < 0.05), but there was no significant difference in age between the CG and the AA-based group (*P* > 0.05). Perforated appendicitis and longer length of stay (LOS) in hospital in the AA group were significantly higher than those in the CG (*P* < 0.05), and there were differences between AA-based groups (*P* < 0.05). No fever in the CG was noted. But the number of fever days in the AA-based groups (SA, PA, and GA) showed an increasing trend (*P* < 0.05), and the difference in thermal spike was not statistically significant (*P* > 0.05).

**Figure 2:**
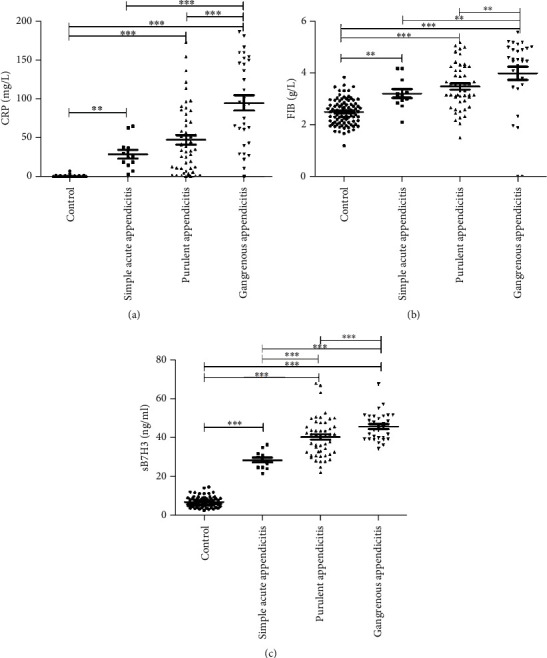
Distribution of CRP, FIB, and sB7H3 in the CG and the AA-based groups: (a) distribution of CRP, (b) distribution of FIB, and (c) distribution of sB7H3. Bars represent median values. Comparisons among the groups were performed using one-way analysis of variance with SNK *t*-test. Statistically significant differences between each patient group are shown as ∗*P* < 0.05, ∗∗*P* < 0.01, and ∗∗∗*P* < 0.001. CG: control group; AA: acute appendicitis; CRP: C-reactive protein; FIB: fibrinogen; sB7H3: soluble B7H3.

The indexes in the AA group, including WBC, CRP, total bilirubin (Tbil), indirect bilirubin (Ibil), direct bilirubin (Dbil), FIB, TNF-*α*, and sB7H3, were significantly higher than those in the CG (*P* < 0.05), and there was a rising trend for these indexes among SA, PA, and GA in turn; however, the differences in CRP, FIB, and sB7H3 among the AA-based groups (SA, PA, and GA) were statistically significant (*P* < 0.05). In addition, the differences of CRP and FIB between the SA and PA groups were not statistically significant (*P* > 0.05) (Figure 2). Additionally, sB7H3 was found as the only marker in children with AA, which has remarkably associated with the degree of histological findings (Figure 1).

There were significant differences in mean corpuscular volume (MCV), red blood cell distribution width (RDW), mean platelet volume (MPV), platelet distribution width (PDW), platelet (PLT), and alkaline phosphatase (ALP) between the AA-based groups (SA, PA, and GA) and the CG, whereas the differences in alanine aminotransferase (ALT), aspartate aminotransferase (AST), and lactate dehydrogenase (LDH) were not statistically significant.

### 3.2. Analysis of Diagnostic Value of Markers for AA

The results of the receiver operating characteristic (ROC) curve analysis and evaluation of the above-mentioned parameters are expressed (Table 2). The markers with high accuracy (0.9 < AUROC ≤ 1) for the diagnosis of AA were sB7H3 and CRP, respectively. The markers with moderate accuracy (0.7 < AUROC ≤ 0.9) in the diagnosis of AA were WBC, Dbil, FIB, Tbil, PDW, Ibil, MCV, and RDW. The markers with low accuracy (AUROC ≤ 0.7) in the diagnosis of AA were TNF-*α* (AUROC, 0.652), PLT (AUROC, 0.302), ALP (AUROC, 0.302), and MPV (AUROC, 0.119).

### 3.3. The Diagnostic Values of sB7H3 for Different Degrees of AA

**Figure 3:**
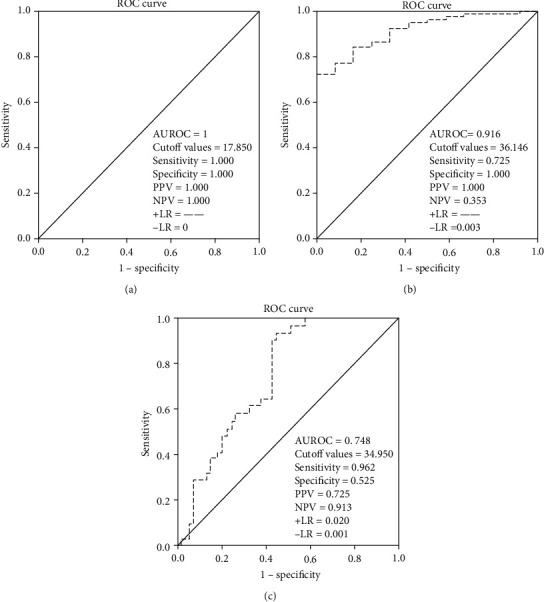
Different ROC curves and parameters for evaluation of sB7H3 for distinguishing different degrees of AA. The ROC curve is drawn by SPSS, and Youden's index is calculated by the corresponding coordinate value on the curve. The maximum value of Youden's index is the ideal cutoff value. (a) The ROC curve and parameters for evaluation of sB7H3 in the nonappendicitis group and the appendicitis group. (b) The ROC curve and parameters for evaluation of sB7H3 in the diagnosis of SA and complex AA (PA+GA). (c) The ROC curve and parameters for evaluation of sB7H3 for the diagnosis of PA and GA. AUROC: area under the curve; PPV: positive predictive value; NPV: negative predictive value; +LR: positive likelihood ratios; -LR: negative likelihood ratios.

The findings showed that sB7H3 had a high diagnostic accuracy for complex AA (PA+GA) (the cutoff value of sB7H3 = 36.146 ng/mL, AUROC = 0.916). However, further results revealed that the diagnostic value of sB7H3 in distinguishing PA from GA in complex AA was not high (the cutoff value of sB7H3 = 34.950 ng/mL, AUROC = 0.748) (Figure 3).

**Figure 4:**
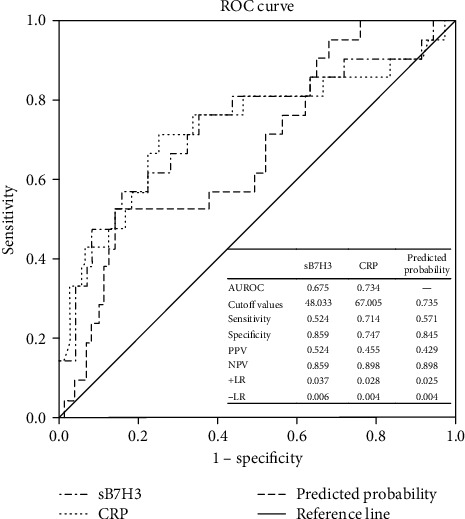
ROC curves and evaluation indexes of sB7H3 and CRP for the diagnosis of appendicitis with perforation. The ROC curve is drawn by SPSS, and Youden's index is calculated by the corresponding coordinate value on the curve. The maximum value of Youden's index is the ideal cutoff value. AUROC: area under the curve; PPV: positive predictive value; NPV: negative predictive value; +LR: positive likelihood ratios; -LR: negative likelihood ratios.

The results of multivariate logistic regression analysis showed that only CRP (*t* = −3.475, *P* = 0.002) and sB7H3 (*t* = −2.309, *P* = 0.023) were statistically different between the nonperforated appendicitis group and the perforated appendicitis group. Further ROC curve analysis found that the AUROC values of CRP and sB7H3 were 0.734 (cutoff values of CRP = 67.005 mg/L, 0.714 SE, 0.747 SP) and 0.675 (cutoff values of sB7H3 = 48.033 ng/mL, 0.524 SE, 0.859 SP), respectively. However, the combination of a CRP level of 67.005 mg/L and a sB7H3 level of 48.033 ng/mL showed 57.1% SE and 84.5% SP, with AUROC of 0.735. The diagnostic performance of CRP and sB7H3 in children with AA was not remarkable, while the diagnostic performance was improved after combination (Table 3 and Figure4).

## 4. Discussion

Despite great familiarity with AA, this disease continues to pose a significant diagnostic challenge for clinicians. This is partially confirmed in very young children whose history is not typical and whose examination results are also unreliable [13]. A delay in the diagnosis of AA could be attributed to nonspecific presentations, overlap of symptoms with a variety of common childhood illnesses, together with inability to express unreliable abdominal examination results in preschool children [14]. Biomarkers can improve the diagnostic performance of AA, especially in children, women at childbearing age, and elderly patients [15]. Traditional biomarkers, e.g., WBC, were found to have a moderate diagnostic performance, while being cost-effective in the diagnosis of AA. In contrast, novel biomarkers were found to be highly expensive, associating with complex compounds, while diagnostic performance can be improved [16].

Moreover, B7H3, also known as CD276, is an immune checkpoint molecule, belonging to the B7-CD28 family. This molecule is associated with costimulatory and coinhibitory functions in regulating T cell responses [17]. Expression of membrane CD276 (mB7H3) has been reported on dendritic cells, monocytes, activated T cells, and various carcinoma cells. The release of sB7H3 from cell surface is mediated by a matrix metalloproteinase and probably regulates B7H3R/B7H3 interactions in vivo [12]. Although no previous study has linked sB7H3 and appendicitis, there is a growing experience to use this marker for detecting other inflammatory conditions. For instance, sB7H3 levels could be significantly elevated in children with bacterial meningitis [10] and Mycoplasma pneumoniae pneumonia (MPP) [18]. Furthermore, Xu et al. [19] analyzed the sB7H3 levels in children with mild MPP and severe MPP and concluded that sB7H3 levels could be helpful for predicting the severity of MPP and investigating treatment efficacy.

In the present study, we included 92 AA patients and 90 inguinal hernia patients as CG to assess the role of sB7H3 levels for predicting the presence and degree of histological findings in children with AA. To our knowledge, this is the first study to evaluate the association between sB7H3 and AA. Although several blood markers can predict AA in children, our results showed that sB7H3 is the only marker, containing a significant correlation with the pathological degree of AA. Furthermore, we demonstrated that sB7H3 has a high diagnostic significance in predicting simple AA and complex AA (PA+GA) in children, and the corresponding values of AUROC were equal to 1.00 (cutoff value of sB7H3 = 17.850 ng/mL) and 0.916 (cutoff value of sB7H3 = 36.146 ng/mL), respectively. However, the diagnostic performance of sB7H3 for distinguishing PA from GA was not found remarkable (cutoff value of sB7H3 = 34.950 ng/mL, AUROC = 0.746). In addition, our results also revealed that only CRP (*t* = −3.475, *P* = 0.002) and sB7H3 (*t* = −2.309, *P* = 0.023) were statistically different between the nonperforated appendicitis group and the perforated appendicitis group, with corresponding AUROC values of 0.734 (cutoff value of CRP = 67.005 mg/L) and 0.675 (cutoff value of sB7H3 = 48.033 ng/mL), respectively. The diagnostic performance of CRP and sB7H3 was not significantly satisfactory to predict perforation of AA in children, while that performance was improved after combination. The AUROC value of 0.735 was achieved after combination of CRP with sB7H3 (57.1% SE and 84.5% SP). Thus, sB7H3 could be a beneficial marker for predicting the presence and severity of AA in children and might be involved in the pathogenesis of AA.

The exact pathogenesis of AA is multifactorial although it still remains elusive. It is irrefutable that obstruction of the lumen is usually present. In preschool children, this obstruction is typically due to lymphoid hyperplasia and less likely due to fecalith, as the appendix contains an excessive amount of lymphoid tissue in the submucosa [14]. Furthermore, the presence of a fecalith causes luminal obstruction, distention, and inflammation of the appendix wall, resulting in suppurative transmural inflammation, ischemia, infarction, and perforation of the appendix [20]. Numerous studies demonstrated that cytokines (e.g., interleukin-6 (IL-6)) [21] or acute phase proteins (e.g., CRP) could be used to predict the AA [1], control the severity of disease, and detect any complications [22]. Therefore, community-acquired intra-abdominal infection and inflammation play a significant role in the development of AA.

The findings of the present study demonstrated that the TNF-*α* level in the AA group was significantly higher than that in the CG, and there was a rising trend of TNF-*α* in the AA-based groups (SA, PA, and GA). Similar to our findings in appendicitis, a comparable result for TNF-*α* was found in a previously reported study [23]. The results of the current research revealed that bacteria, endotoxin, and other factors may cause an increase in the release of a number of cytokines (e.g., TNF-*α*) in AA. In addition, the results showed that sB7H3 and TNF-*α* both have a rising trend in the AA-based groups (SA, PA, and GA). Thus, the correlation between sB7H3 and TNF-*α* was investigated here. The findings disclosed positive correlations between plasma sB7H3 levels and TNF-*α* levels in patients with AA (*y* = 1.3*x* + 9.85, *R*^2^ = 0.087, *P* < 0.001). However, no correlations were found between plasma sB7H3 levels and TNF-*α* levels in patients with SA (*P* > 0.05), PA (*P* > 0.05), and GA (*P* > 0.05). These data proved the existence of a relationship between sB7H3 with TNF-*α*. In our previous study, we observed that substantial amounts of sB7H3 were released from freshly isolated human monocytes upon stimulation with TNF-*α* compared with naive cells [11]. This evidence could explain the underlying mechanisms being responsible for the significantly elevated plasma sB7H3 levels observed in patients with AA.

The diagnostic accuracy of markers for the diagnosis of AA was found to be more accurate in the present study compared with previous studies, in which the AUROC values for WBC and CRP were 0.895 and 0.983, respectively (Table 2). A number of studies have taken nonspecific abdominal pain (NSAP) patients as CG into consideration. For instance, Oikonomopoulou et al. [24] selected 185 non-AA cases as CG. The CG included NSAP (151, 81.6%), followed by mesenteric lymphadenitis (16, 8.6%), ileitis (4, 2.2%), acute gastroenteritis (5, 2.7%), pneumonia (2, 1.1%), streptococcal pharyngitis (1, 0.5%), influenza type B virus (1, 0.5%), constipation (1, 0.5%), and intussusception (1, 0.5%). The AUROC values of leukocytes and CRP were 0.84 and 0.7, respectively. In another study, Kaiser et al. [7] recruited 25 NASP children who improved under conservative treatment and did not require surgical intervention and served as CG. The specific causes of NSAP were gastroenteritis (18, 72%), constipation (4, 16%), and abdominal cramps based on food intolerance (3, 12%). The AUROC values of leukocytes and CRP were 0.711 and 0.619, respectively. As a nonspecific response of the body, raised white blood cells and CRP levels are frequent in patients with acute and chronic gastroenteritis accompanied by vomiting, abdominal pain, dehydration, and other serious symptoms. This condition may be misdiagnosed as AA in several cases. Moreover, respiratory and urinary tract infections, as the causes of acute abdominal pain in children [25–27], may often accompany by elevated levels of leukocytes and CRP. However, these conditions do not occur in selective hernia surgery.

In addition, a number of studies on appendicitis have selected negative appendectomy patients as CG. However, Dubrovsky et al. [28] demonstrated that negative appendicitis is associated with greater morbidity, longer LOS, higher complication rate, and higher cost compared with nonperforated appendicitis. In addition, they identified a total of 156,660 nonincidental inpatient appendectomies from 2005 to 2011. They observed an overall decrease in the rate of both negative appendicitis (3.3% in 2005 to 1.8% in 2011, *P* < 0.01) and perforated appendicitis (27.1% in 2005 to 25.0% in 2011, *P* < 0.01). Similar results were noted in studies conducted by Chinese scholars. For instance, Jin et al. [29] expressed that the negative appendectomy rate was 1.53% (31/2015) in Chongqing (China). There were two patients with normal appendix according to the results of histology in that study. However, the negative appendectomy rate in a pediatric appendicitis study with a large sample size reached 10.75% (37/344) in Turkey [30] and 15.54% (186/1197) in three pediatric centers performed in Canada, Australia, and the UK [22]. Dubrovsky et al. [28] demonstrated higher proportion of gastrointestinal complications (obstruction or C. difficile infection) and respiratory complications (e.g., atelectasis or pneumonia) in negative appendicitis patients than in nonperforated appendicitis patients. Thus, patients with negative appendectomy rate may highly have higher levels of leukocytes and CRP than healthy individuals.

Therefore, compared with NSAP and negative appendectomy, we selected nonemergency inguinal hernia as CG, and the diagnostic efficiency of markers was relatively high. A recent study conducted by Sarsu et al. [6] also selected healthy controls and demonstrated that in diagnosis of complicated AA, AUROC for fecal lactoferrin, serum CPR, and serum HMGB-1 were determined as 1.00 and the cutoff level was determined as 25 *μ*g/g feces, 670 ng/mL, and 30 ng/mL, respectively. In differential diagnosis of uncomplicated and complicated AA, the most accurate parameter was fecal lactoferrin with an AUROC of 0.977.

Studies on the role of hyperbilirubinemia in appendicitis were mainly concentrated on adult patients. In the present study, the bilirubin level in the AA group was significantly higher than that in the CG. Furthermore, our results demonstrated that the Dbil level had a higher SE and AUROC than the Tbil and Ibil levels for AA. These results were in agreement with Eren et al.'s achievements [31], in which hyperbilirubinemia, especially with elevated direct bilirubin levels, was elevated significantly in gangrenous/perforated appendicitis. However, there were no significant differences in Tbil, Dbil, and Ibil levels between the nonperforated appendicitis group and the perforated appendicitis group in the present study. This was found to be consistent with a meta-analysis conducted by Silva et al. [32], who demonstrated that the diagnostic value of hyperbilirubinemia cannot merely predict acute perforated appendicitis. Thus, hyperbilirubinemia may be a moderate marker to predict AA in children, while that cannot merely predict perforation.

In the present study, significantly higher values of PDW, MCV, and RDW were noted in children with AA. The AUROC values for PDW, MCV, and RDW were 0.776, 0.724, and 0.711, respectively. In addition, RDW had the highest SE of 0.902 and the lowest SP of 0.456. Furthermore, the results of the current research demonstrated that RDW values increased with progress of severity of appendicitis, while the difference was not statistically significant. There were no significant differences in PDW, MCV, RDW, and MPV between the nonperforated appendicitis group and the perforated appendicitis group. In agreement with the current research, another study [30] related to RDW on children who suspected of having appendicitis revealed that it might be precious for diagnosing AA in children, rather than utilizing for predicting perforation. In contrast to studies conducted on children, Boshnak et al. [33] reported that the PDW level in the positive appendectomy group was significantly higher than that in the negative appendectomy group. Significantly higher RDW level was only found in patients with AA who developed complications compared with those without complications. The diagnostic performance of RDW and PDW for diagnosing AA was not considerable; the AUROC values for PDW were determined as 0.696. Hence, increased levels of PDW and RDW combined with elevated WBC and neutrophil counts may be as advantageous for diagnosing cases suspected of having AA.

In the present study, the FIB level in patients with AA was significantly higher than that in the CG, and FIB level increased with progress of severity of appendicitis. However, there was no significant difference in the FIB level between SA and PA. Serum FIB level has been studied for diagnosing AA in a limited number of studies. Menteş et al. [34] investigated the diagnostic role of FIB in AA and found that the serum FIB level (accuracy, 68.16%) had a similar diagnostic value to WBC (accuracy, 72.14%). This result was comparable to that achieved in the present research. Our results showed that FIB and WBC both had moderate accuracy for diagnosing AA.

Perforated appendicitis is associated with short- and long-term complications, including peritonitis, sepsis, bowel intestinal obstruction, abscess formation, and fertility problems. Furthermore, the rate of perforated appendicitis is higher in children than in adults, especially in younger children (<5 years) [35, 36]. In the current study, the perforated rate of AA was 22.83% (21/92). Furthermore, there were significant differences in the levels of CRP and sB7H3 between the nonperforated appendicitis group and the perforated appendicitis group, in which the values of AUROC were 0.734 and 0.675, respectively. With combination of CRP and sB7H3, the value of AUROC in predicting perforated appendicitis was determined as 0.735. Although the younger age, the longer LOS, the longer duration of fever, and the higher levels of markers (RDW, NR, Dbil, FIB, and TNF-*α*) were found in the current research, the differences were not statistically significant. Buyukbese et al. [37] retrospectively studied 317 children who underwent appendectomy and found that the rate of complicated AA was 24.92% (79/317), and the CRP had the highest diagnostic value in predicting complicated appendicitis (AUROC, 0.887 L). Kim et al. [38] evaluated the predictive values of delta neutrophil index (DNI) and myeloperoxidase index (MPXI) in 105 children with AA and found that the complicated rate was 27.6% (29/105) and the CRP had the maximum value of AUROC (0.840) when cutoff was 40 mg/L. Being consistent with the present study, Kaiser et al. [7] also demonstrated that a combination of different inflammation markers may associate with higher AUROC value to predict complicated appendicitis.

As previously described, sB7H3 as a new biomarker has advantages in diagnostic efficiency over the old biomarkers (CRP, WBC, Dbil, etc.). However, the clinical application is inconvenient and more expensive now. If a commercial company intervenes later, the convenience and cost will definitely improve significantly.

The current study was limited by a relatively limited number of patients with AA, especially patients with SA. Nevertheless, the serum level of sB7H3 was only examined at the time of admission. Additionally, the time from the onset of symptoms to diagnosis of AA in children was not recorded. Therefore, further studies should be conducted on a larger sample size at different time points with inclusion of data of AA, in order to accurately assess the diagnostic value of sB7H3 for AA in children.

## 5. Conclusions

In summary, we noted significantly elevated sB7H3 level in children with AA, and sB7H3 levels remarkably associated with the degree of histological findings of the appendix. Hence, sB7H3 could be used as a precious marker to predict the presence of AA and complex AA in children. However, the diagnostic value of sB7H3 to predict gangrenous/perforated appendicitis was not notable. The combination of sB7H3 and CRP might improve the prediction of perforated appendicitis.

## Figures and Tables

**Table 1:** Demographic data and clinical characteristics of AA and control subjects.

Parameters	Control group (*N* = 90)	AA patients (*n* = 92)	AA-based groups (*N* = 92)			*H*/*F*/*χ*^2^	*P*
			SA (*N* = 12)	PA (*N* = 49)	GA (*N* = 31)		
Sex (M, %)	54 (60.00%)	62 (67.39%)	7 (58.33%)	36 (73.47%)	19 (61.29%)	2.871	0.412
Age (mean ± SD, years)	6.26 ± 2.48	7.38 ± 3.52^###^	6.5 ± 1.78	7.80 ± 3.83	7.06 ± 3.50	4.830	0.185
Perforated appendicitis (*N*, %)	0 (0%)	21 (22.82%)^###^	0 (0%)	6 (12.24%)	15 (48.39%)	18.161	≤0.001
LOS (mean ± SD, days)	4.33 ± 0.91	8.34 ± 3.76^###^	8.00 ± 2.09	7.65 ± 2.14	9.55 ± 5.63	122.480	≤0.001∗
Fever days (mean ± SD, days)	0	2.23 ± 1.86^###^	1.17 ± 0.84	2.06 ± 1.78	2.90 ± 2.06	141.087	≤0.001∗
Thermal spike (mean ± SD, °C)	0	38.69 ± 0.67^###^	38.42 ± 0.53	38.60 ± 0.64	38.90 ± 0.71	2.907	0.060
WBC (mean ± SD, 1000/*μ*L)	9.38 ± 2.92	16.72 ± 5.51^###^	15.04 ± 4.43	16.96 ± 5.30	17.00 ± 6.22	85.335	≤0.001∗
MCV (mean ± SD, fL)	78.99 ± 5.44	82.14 ± 4.83	82.08 ± 2.94	82.69 ± 4.42	81.29 ± 5.93	6.153	0.001∗
RDW (mean ± SD, %)	13.46 ± 1.66	14.10 ± 1.21	13.90 ± 0.84	14.12 ± 1.12	14.16 ± 1.48	3.053	0.030∗
MPV (mean ± SD, fL)	9.90 ± 0.80	8.06 ± 1.25^##^	7.75 ± 1.09	8.14 ± 1.30	8.07 ± 1.25	79.596	≤0.001∗
PDW (mean ± SD, %)	10.92 ± 1.58	13.06 ± 2.99^##^	12.51 ± 0.60	13.51 ± 3.20	12.57 ± 3.14	41.492	≤0.001∗
CRP (mean ± SD, mg/L)	0.42 ± 0.91	61.21 ± 52.18^###^	28.92 ± 19.54	47.57 ± 43.87	95.27 ± 55.83	130.964	≤0.001∗
PLT (mean ± SD, 1000/*μ*L)	320.1 ± 84.70	257.29 ± 78.63	260.75 ± 39.54	257.69 ± 86.67	255.32 ± 78.45	8.880	≤0.001∗
ALT (mean ± SD, U/L)	16.43 ± 6.41	15.37 ± 13.72	12.63 ± 3.44	14.77 ± 12.14	17.37 ± 17.37	0.818	0.486
AST (mean ± SD, U/L)	32.84 ± 10.45	32.52 ± 12.17	34.67 ± 8.39	32.03 ± 12.22	32.45 ± 13.51	0.184	0.907
ALP (mean ± SD, U/L)	262.81 ± 115.87	205.20 ± 55.31^##^	199.34 ± 17.23	213.42 ± 55.60	194.48 ± 63.10	5.315	0.021∗
Tbil (mean ± SD, *μ*mol/L)	7.07 ± 5.85	12.98 ± 9.29^##^	10.33 ± 1.07	12.57 ± 7.91	14.66 ± 12.45	59.41	≤0.001∗
Ibil (mean ± SD, *μ*mol/L)	4.94 ± 4.50	8.64 ± 7.40^#^	6.92 ± 1.14	8.42 ± 6.60	9.67 ± 9.66	40.256	≤0.001∗
Dbil (mean ± SD, *μ*mol/L)	2.13 ± 1.41	4.31 ± 2.57^###^	3.41 ± 0.57	4.11 ± 2.00	5.00 ± 3.56	76.552	≤0.001∗
LDH (mean ± S D, U/L)	297.53 ± 99.64	284.81 ± 99.10	254.91 ± 21.28	283.61 ± 92.37	298.28 ± 124.20	3.787	0.285
FIB (mean ± SD, g/L)	2.50 ± 0.49	3.61 ± 1.08^###^	3.21 ± 0.59	3.48 ± 0.86	3.99 ± 1.41	68.397	≤0.001∗
TNF-*α* (mean ± SD, pg/mL)	16.88 ± 5.18	63.71 ± 109.86^###^	55.22 ± 33.73	56.13 ± 122.79	78.97 ± 108.19	28.930	≤0.001∗
sB7H3 (mean ± SD, ng/mL)	6.58 ± 2.38	40.29 ± 10.17^###^	28.15 ± 4.50	40.02 ± 10.24	45.43 ± 7.24	143.057	≤0.001∗

AA: acute appendicitis; SA: simple appendicitis; PA: purulent appendicitis; GA: gangrenous appendicitis. Asterisks indicate that the reorganization data is nonnormal distribution, and the Kruskal Wallis test is used to analyze the differences between groups. The mean of other groups was compared by one-way ANOVA/Student's *t*-test for independent samples, and the rate was compared by the chi-square test. Statistically significant differences between AA patients and control group are shown in column 3 as ^#^*P* < 0.05, ^##^*P* < 0.01, and ^###^*P* < 0.001. Statistically significant differences between four groups are shown in the last column. LOS: length of stay in hospital; WBC: white blood cell; MCV: mean corpuscular volume; RDW: red blood cell distribution width; MPV: mean platelet volume; PDW: platelet distribution width; CRP: C-reactive protein; PLT: platelet; ALT: alanine aminotransferase; AST: aspartate aminotransferase; ALP: alkaline phosphatase; Tbil: total bilirubin; Ibil: indirect bilirubin; Dbil: direct bilirubin; LDH: lactate dehydrogenase; FIB: fibrinogen; TNF-*α*: tumor necrosis factor-*α*.

**Table 2:** The results of the receiver operating characteristic (ROC) curve analysis and evaluation of the above-mentioned parameters of blood markers for AA.

Parameters	Cutoff values	AUROC	Sensitivity (SE)	Specificity (SP)	PPV	NPV	+LR	-LR
sB7H3 (ng/mL)	17.850	1	1.000	1.000	1.000	1.000	—	0.000
CRP (mg/L)	1.905	0.983	0.935	0.967	0.966	0.935	0.280	0.001
WBC (1000/*μ*L)	12.495	0.895	0.826	0.878	0.874	0.832	0.068	0.002
Dbil (*μ*mol/L)	2.45	0.874	0.891	0.778	0.804	0.875	0.040	0.001
FIB (g/L)	3.11	0.845	0.728	0.911	0.893	0.766	0.082	0.003
Tbil (*μ*mol/L)	8.315	0.831	0.761	0.789	0.787	0.763	0.036	0.003
PDW (%)	12.05	0.776	0.674	0.800	0.775	0.706	0.034	0.004
Ibil (*μ*mol/L)	6.875	0.772	0.522	0.900	0.842	0.648	0.052	0.005
MCV (fL)	80.5	0.724	0.761	0.589	0.654	0.707	0.019	0.004
RDW (%)	12.85	0.711	0.902	0.456	0.629	0.820	0.017	0.002

The ROC curve is drawn by SPSS, and Youden's index is calculated by the corresponding coordinate value on the curve. The maximum value of Youden's index is the ideal cutoff value. AUROC: area under the curve; PPV: positive predictive value; NPV: negative predictive value; +LR: positive likelihood ratios; -LR: negative likelihood ratios; CRP: C-reactive protein; WBC: white blood cell; Dbil: direct bilirubin; FIB: fibrinogen; Tbil: total bilirubin; PDW: platelet distribution width; MCV: mean corpuscular volume; PDW: platelet distribution width; Ibil: indirect bilirubin; MCV: mean corpuscular volume; RDW: red blood cell distribution width.

**Table 3:** Comparing patients' demographic data and clinical characteristics between the perforated appendicitis group and the nonperforated appendicitis group.

Parameters	Nonperforated appendicitis *N* = 71 (PA = 43, GA = 28)	Perforated appendicitis *N* = 21 (PA = 6, GA = 15)	*t*/*Zχ*^2^	*P* value
Sex (M, %)	46 (64.79%)	16 (76.19%)	0.959	0.328
Age (mean ± SD, years)	7.59 ± 3.54	6.67 ± 3.43	1.058	0.293
LOS (mean ± SD, days)	7.77 ± 2.11	10.24 ± 6.63	-1.679	0.108
Duration of fever (mean ± SD, days)	2.06 ± 1.74	2.81 ± 2.16	-1.647	0.103
Thermal spike (mean ± SD, °C)	38.63 ± 0.68	38.87 ± 0.63	-1.406	0.163
WBC (mean ± SD, 1000/*μ*L)	16.89 ± 5.71	16.15 ± 4.87	-0.542	0.589
MCV (mean ± SD, fL)	82.13 ± 4.97	82.19 ± 4.46	-0.053	0.958
RDW (mean ± SD, %)	14.10 ± 1.09	14.11 ± 1.58	-0.008	0.993
MPV (mean ± SD, fL)	8.10 ± 1.31	7.96 ± 1.04	0.447	0.656
PDW (mean ± SD, %)	13.25 ± 2.76	12.44 ± 3.66	1.093	0.277
CRP (mean ± SD, mg/L)	49.80 ± 43.34	99.78 ± 61.54	-3.475	0.002∗
PLT (mean ± SD, 1000/*μ*L)	255.65 ± 72.74	262.86 ± 97.85	-0.367	0.714
ALT (mean ± SD, U/L)	14.06 ± 10.70	19.79 ± 20.72	-1.219	0.214
AST (mean ± SD, U/L)	31.63 ± 12.04	35.52 ± 42.43	-1.290	0.200
ALP (mean ± SD, U/L)	207.91 ± 55.10	196.04 ± 56.37	0.863	0.391
Tbil (mean ± SD, *μ*mol/L)	13.03 ± 9.66	12.84 ± 8.14	0.083	0.934
Ibil (mean ± SD, *μ*mol/L)	8.82 ± 7.98	8.05 ± 5.05	0.414	0.680
Dbil (mean ± SD, *μ*mol/L)	4.21 ± 2.26	4.69 ± 3.47	-0.749	0.456
LDH (mean ± SD, U/L)	274.47 ± 80.04	319.74 ± 143.54	-1.383	0.180
FIB (mean ± SD, g/L)	3.61 ± 0.90	3.63 ± 1.56	-0.037	0.971
TNF-*α* (mean ± SD, pg/mL)	58.85 ± 94.60	80.13 ± 152.39	-0.778	0.439
sB7H3 (mean ± SD, ng/mL)	38.99 ± 10.12	44.69 ± 9.29	-2.309	0.023∗

Asterisks (∗) indicate that the reorganization data is nonnormal distribution, and rank sum test is used to analyze the differences between groups. LOS: longer length of stay in hospital; WBC: white blood cell; MCV: mean corpuscular volume; RDW: red blood cell distribution width; MPV: mean platelet volume; PDW: platelet distribution width; CRP: C-reactive protein; PLT: platelet; ALT: alanine aminotransferase; AST: aspartate aminotransferase; ALP: alkaline phosphatase; Tbil: total bilirubin; Ibil: indirect bilirubin; Dbil: direct bilirubin; LDH: lactate dehydrogenase; FIB: fibrinogen; TNF-*α*: tumor necrosis factor-*α*.

## Data Availability

The data used to support the findings of this study are available from the corresponding author upon request.
